# Housing Insecurity, Incident Geriatric Conditions, and Mortality in Community-Living Older Persons

**DOI:** 10.1001/jamanetworkopen.2026.9335

**Published:** 2026-05-01

**Authors:** Yi Wang, Kendra Davis-Plourde, Brent Vander Wyk, Lucero G. Paredes, Thomas M. Gill, Robert D. Becher

**Affiliations:** 1 Department of Internal Medicine, Yale School of Medicine, New Haven, Connecticut; 2 Department of Biostatistics, Yale School of Public Health, New Haven, Connecticut; 3 Department of Surgery, Maine Medical Center, Portland; 4 Department of Surgery, Yale School of Medicine, New Haven, Connecticut

## Abstract

**Importance:**

Housing insecurity is a key social determinant of health, yet its association with health outcomes among older persons has been understudied.

**Objective:**

To examine the associations between housing insecurity and the development of frailty, disability, and dementia, as well as mortality.

**Design, Setting, and Participants:**

This prospective cohort study was based on data from the National Health and Aging Trends Study (NHATS) from 2015 to 2020. Data analysis was conducted from August 2024 to February 2026. Participants were community-living persons aged 65 years or older in the contiguous US.

**Exposures:**

Three forms of housing insecurity derived from the NHATS annual survey: poor housing affordability, poor housing quality, and poor neighborhood quality.

**Main Outcomes and Measures:**

The primary outcomes were time to onset of frailty, disability, and dementia, and time to death over 5 years. Geriatric conditions were obtained from the NHATS annual survey, and all-cause mortality from linked Medicare records. Discrete cause-specific hazards models accounting for the competing risk of death (equivalent to multinomial logistic regression) were used to estimate relative risk ratios (RRRs) for geriatric conditions, and time-varying Cox regression models were used to estimate hazard ratios (HRs) for mortality.

**Results:**

Among the 7499 participants (mean [SD] age, 78.2 [7.8] years; 4335 [55.3%] female), after adjustment for age, sex, race and ethnicity, education, Medicaid eligibility, household income, smoking status, and comorbidity, poor housing affordability was significantly associated with higher risks of frailty (RRR, 1.23; 95% CI, 1.01-1.49), disability (RRR, 1.24; 95% CI, 1.01-1.54), dementia (RRR, 1.37; 95% CI, 1.11-1.69), and mortality (HR, 1.51; 95% CI, 1.34-1.70). Similarly, poor housing quality was significantly associated with higher risks of frailty (RRR, 1.30; 95% CI, 1.04-1.62), disability (RRR, 1.33; 95% CI, 1.13-1.57), and mortality (HR, 1.15; 95% CI, 1.01-1.32), but not dementia (RRR, 1.16; 95% CI, 0.90-1.49). Poor neighborhood quality was not associated with any outcome in the adjusted analyses. The adjusted risk differences ranged from 1.9 percentage points (95% CI, 0.2-3.1 percentage points) for housing quality with mortality to 11.1 percentage points (95% CI, 7.9-14.3 percentage points) for housing affordability with disability.

**Conclusions and Relevance:**

In this cohort study of community-living older US persons, poor housing affordability was associated with higher risks of frailty, disability, dementia, and mortality, and poor housing quality was associated with higher risks of frailty, disability, and mortality. These findings highlight housing insecurity as a clinically relevant social determinant of health among older persons.

## Key Points

**Question:**

Is housing insecurity associated with incident geriatric conditions and mortality in community-living older persons?

**Findings:**

In this cohort study of 7499 community-living older US persons, poor housing affordability was significantly associated with higher subsequent risks of frailty, disability, dementia, and mortality, while poor housing quality was associated with higher risks of frailty, disability, and mortality, but not dementia. Neighborhood quality was not associated with any outcome.

**Meaning:**

These findings suggest that poor housing affordability and poor housing quality are important social determinants of health associated with incident geriatric conditions and mortality in older persons.

## Teaser

This cohort study investigates the associations between housing insecurity and incident geriatric conditions and mortality in community-living older persons.

## Introduction

Housing insecurity is a key and potentially modifiable social determinant of health (SDOH).^1,2,3^ Prior research has shown that housing insecurity—defined by a lack of housing affordability, quality, and stability—is associated with several adverse health outcomes, including poor self-rated health, psychological distress, and increased emergency department visits.^4,5^ Despite growing interest in housing insecurity, relatively few studies have examined its association with health outcomes among persons aged 65 years or older. Because more than 1 in 3 community-living older individuals in the US are estimated to be housing insecure,^2^ this represents an important knowledge gap.

For older persons, living with and experiencing housing insecurity may have particularly negative downstream consequences for at least 2 reasons. First, housing is costly for many older persons,^6^ whose incomes are typically fixed or decline with age,^7,8^ while their health care needs and medical expenses increase. For example, persons aged 65 years or older spend nearly twice as much on out-of-pocket health care as those aged 18 to 64 years, and 4 times as much as those younger than 18 years.^9^ Lack of affordable housing may further constrain their ability to pay for adequate nutrition, medications, medical care, and other essential health-related expenses, potentially leading to poorer health outcomes.^2,10^ Second, as physiological reserves diminish with age, older persons are sensitive to environmental stressors.^11,12^ As a result, those living in poor-quality housing (eg, the presence of broken windows) or in poor-quality neighborhoods (eg, the presence of litter or trash on the streets) may be especially vulnerable to the adverse health outcomes associated with such exposures.

Despite the heightened housing-related vulnerabilities of older persons, little research has evaluated whether specific forms of housing insecurity are associated with clinically meaningful outcomes in this population. For older persons, mortality serves as a key and easily interpretable indicator of overall health, while geriatric conditions—such as frailty, disability, and dementia—are indicators of health and well-being that affect quality of life.^13,14^ Understanding whether and how key forms of housing insecurity are associated with these outcomes has important implications for clinical practice and may help guide public health and housing policy interventions targeted to aging populations. This is important because housing insecurity is potentially modifiable through specific policy interventions, including public housing, rental assistance, and housing rehabilitation programs.^15,16,17^ In this study, we used high-quality data from a nationally representative sample of community-living older US persons to evaluate the associations between 3 forms of housing insecurity—poor housing affordability, poor housing quality, and poor neighborhood quality—and the development of 3 geriatric conditions (frailty, disability, and dementia) and mortality over a 5-year follow-up period.

## Methods

### Study Design and Population

**Figure 1.**
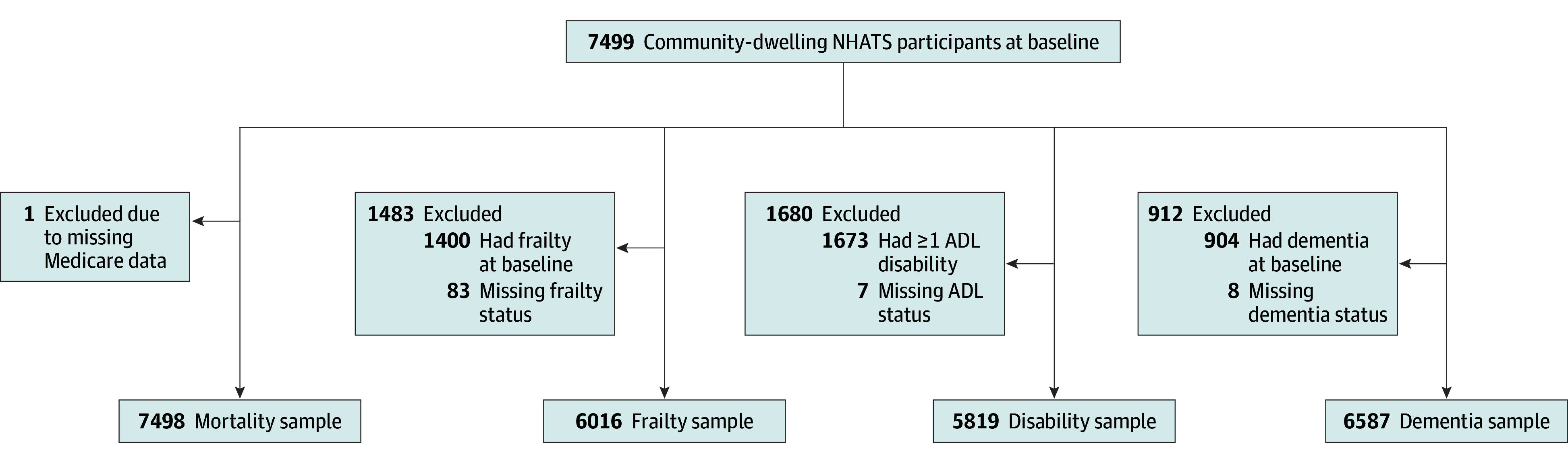
Flow Diagram of Sample Selection Process Samples represent unweighted values. ADL indicates activities of daily living; NHATS, National Health and Aging Trends Study.

This cohort study used data from the National Health and Aging Trends Study (NHATS), a nationally representative cohort study of Medicare beneficiaries aged 65 years or older.^18^ The original cohort was established in 2011, with a subsequent replenishment on September 30, 2014—referred to as the 2015 cohort—to maintain representation of the older Medicare population in the contiguous US. Detailed descriptions of the study’s design and sampling methods are available elsewhere.^18,19^ For this analysis, we used contemporary data from the 2015 cohort, which allows for the inclusion of community-living participants residing in assisted living facilities,^18^ as prior to 2015 (round 5) housing cost data (a key component of housing affordability) were not collected. We selected a 5-year follow-up period because data on housing quality and neighborhood quality were not collected during round 10 due to the COVID-19 pandemic. This study focused on participants living in settings other than nursing homes (ie, community living), yielding a baseline round 5 sample. For the analysis of geriatric conditions, participants with the respective conditions at baseline were excluded to ensure that incident cases were evaluated. Figure 1 shows the assembly of the analytic sample for each outcome.

The NHATS data have been linked to Medicare records, allowing us to more accurately identify deaths during the follow-up period.^20^ NHATS was approved by Johns Hopkins University institutional review board, and all participants provided written informed consent. The current study, which was approved by the Yale Human Investigation Committee, followed the Strengthening the Reporting of Observational Studies in Epidemiology (STROBE) reporting guideline for cohort studies.^21^

### Assessment of Housing Insecurity

Three forms of housing insecurity were assessed, using data from the NHATS annual survey: poor housing affordability, poor housing quality, and poor neighborhood quality.^2^ Poor housing affordability was characterized as the presence of housing cost burden, defined as spending at least 30% of the monthly income of the participant and their cohabiting spouse or partner on housing costs, including self-reported rent or mortgage payments.^10^ For income, missing values were imputed and provided by NHATS.^22^ Poor housing quality, determined from interviewer-recorded observations recorded on 2 sections of the NHATS environmental checklist, was defined as the presence of any of 5 interior indicators (peeling or flaking paint, pests, broken furniture or lamps, flooring in need of repair, or other tripping hazards) or 5 exterior indicators (broken or boarded-up windows, crumbling foundations or open holes, missing bricks or siding, roof problems, or uneven walking surfaces or broken steps leading to the home). Poor neighborhood quality, assessed using a third section of the environmental checklist, was defined by the presence of any of the listed indicators: litter, broken glass, or trash on sidewalks and streets; graffiti on buildings and walls; or vacant or abandoned houses or storefronts. The percentages of missing data were 7.3% (498 individuals) for housing affordability, 8.7% (568 individuals) for housing quality, and 2.4% (157 individuals) for neighborhood quality.

### Outcomes

The outcomes included (1) time to onset of frailty, disability, and dementia and (2) time to death, over the 5-year follow-up period. The 3 geriatric conditions were determined from the NHATS annual surveys through 2020. The definitions of the geriatric conditions are well-established and widely used in prior NHATS research.^23,24,25^ Frailty was assessed using the Fried phenotype, which includes 5 criteria: unintentional weight loss, slowness, weakness, low physical activity, and exhaustion. Participants who met 3 criteria were classified as frail.^23^ Due to the COVID-19 pandemic, grip strength and walking speed could not be assessed; therefore, frailty in 2020 was defined as meeting all 3 of the remaining criteria.^25^ Disability was defined as requiring assistance from another person to complete 1 or more of 7 activities of daily living: getting cleaned up, using the toilet, dressing, eating, getting around inside, going outside, and getting out of bed.^25^ On the basis of the NHATS-validated algorithm, participants were classified as having probable dementia (hereafter, dementia) if they scored 1.5 SD below the mean of self-respondents in at least 2 cognitive domains (memory, orientation, or executive function), had a self-reported or proxy-reported dementia diagnosis, or met AD8 (the 8-item dementia screening scale) criteria in the absence of a reported diagnosis.^24^ All-cause mortality was determined using linked Medicare records through December 31, 2020.

### Covariates

Covariates were chosen on the basis of an evidence-based conceptual framework (eFigure 1 in Supplement 1). All covariates were obtained from the NHATS baseline survey, including demographic characteristics, socioeconomic status, smoking status (current smoker vs not), and comorbidity. The demographic characteristics included sex, age group (5-year categories from 65 to 90 years old), and self-reported race and ethnicity. Data on race and ethnicity are included in this study because they are important social determinants of health and potential confounders of the association between housing insecurity and health outcomes. The socioeconomic status included educational attainment, Medicaid eligibility, and household income.^26,27^ Comorbidity was measured as the count of 9 self-reported, physician-diagnosed chronic conditions, including heart attack, heart disease, high blood pressure, arthritis, osteoporosis, diabetes, lung disease, stroke, and cancer.^28^

### Statistical Analysis

To account for the complex survey design of the NHATS, all analyses incorporated the analytic weights, strata, and clustering elements,^18,19^ unless otherwise stated. Because the prevalence of the 3 forms of housing insecurity varied over time (eTables 1-3 in Supplement 1), all analyses treated housing insecurity as time-varying. Descriptive characteristics were calculated at baseline.

We used extended Kaplan-Meier curves^29,30^ to visualize cumulative incidence of geriatric conditions and mortality according to housing insecurity; for mortality, we collapsed intervals as needed to ensure that each contained at least 11 unweighted deaths to meet the requirements of the National Institute on Aging Data Linkage Program.^31^ We used discrete cause-specific hazards models accounting for the competing risk of death (equivalent to multinomial logistic regression) to estimate relative risk ratios (RRRs) and 95% CIs for geriatric conditions,^32,33,34^ and time-varying Cox regression models to estimate hazard ratios (HRs) and 95% CIs for mortality.^35^ Participants who neither developed the respective geriatric condition nor died were censored at the date of their last interview, and nondecedents in the mortality analysis were censored on December 31, 2020. Four sequentially adjusted models were fitted to determine how estimates changed with the inclusion of additional covariate sets. E-values were subsequently calculated to quantify the minimum strength of unmeasured confounding needed to fully explain the observed associations.^36,37^ Adjusted risk differences (RDs) were also calculated to quantify the corresponding absolute differences between groups. Statistical significance was defined as a 95% CI that excluded 1 for RRRs and HRs and 0 for RDs.

We conducted 2 sensitivity analyses. First, to account for missing data, we repeated the fully adjusted models using multiple imputation by chained equations (10 imputations), incorporating all covariates.^38,39^ Second, to further account for area-level disadvantage, we included the Geriatric Index of County-Level Multi-Dimensional Contextual Disadvantage (GERiCounty)^25^ as an additional covariate in the fully adjusted models. Because linkage of GERiCounty with Medicare data are not permitted,^31^ mortality data for this analysis were obtained from the NHATS Sensitive Files. All analyses were conducted using SAS statistical software version 9.4 (SAS Institute), R statistical software version 4.4.2 (R Project for Statistical Computing), and Stata statistical software version 17.0 (StataCorp). Data were analyzed from August 2024 to February 2026.

## Results

**Figure 2.**
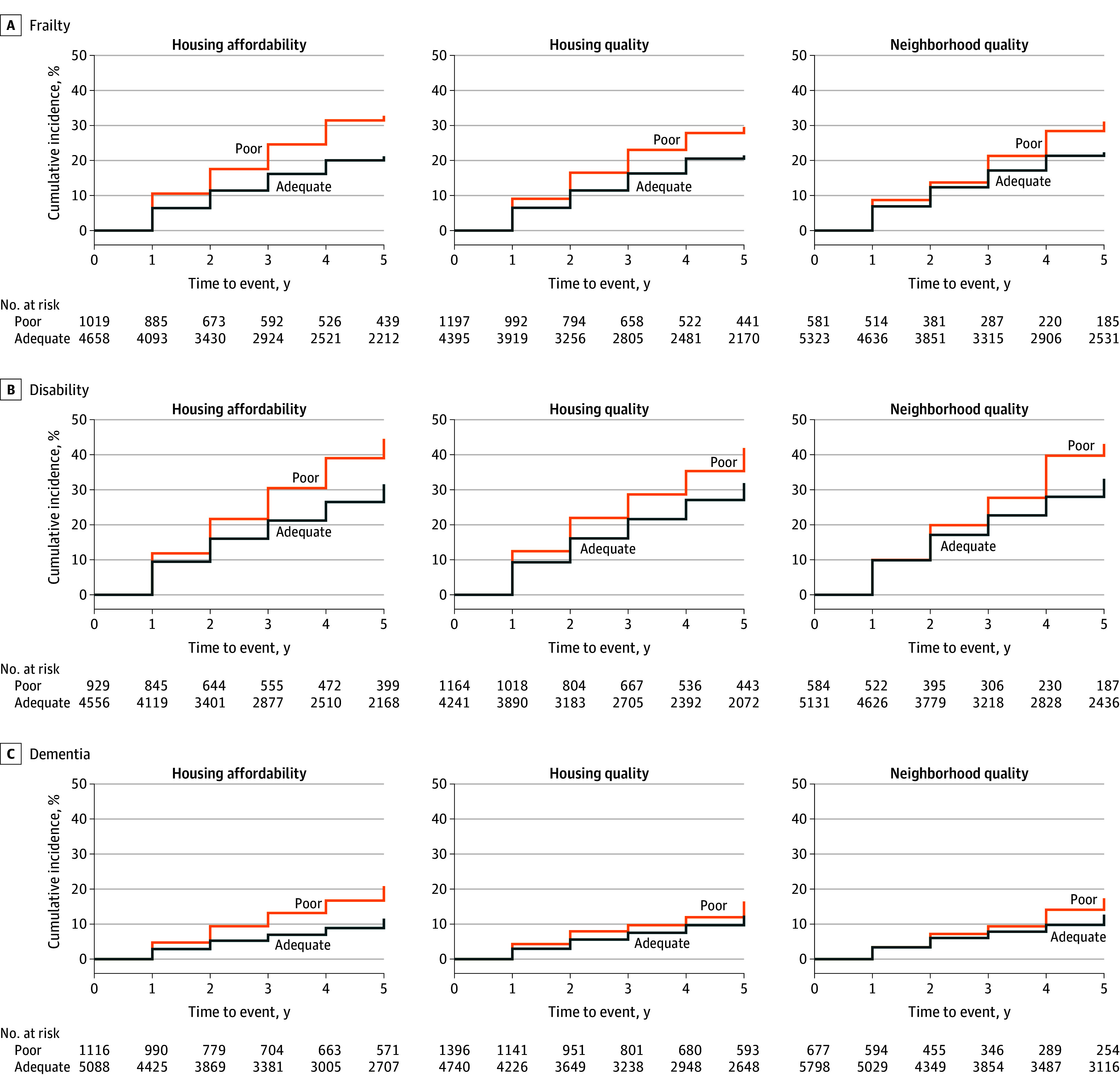
Curves of Cumulative Incidence of Geriatric Conditions Over 5 Years for 3 Forms of Housing Insecurity Analytic weights provided by National Health and Aging Trends Study were applied to account for differential probabilities of selection and nonresponse. The number at risk represents unweighted values.

Among the 7499 community-living persons (representing 40 728 543 survey-weighted persons) included in the study, the mean (SD) age was 78.2 (7.8) years, with 4335 (55.3%) being female. A total of 1600 (16.7%) had a less than high school education, and 1063 (12.1%) were eligible for Medicaid. The baseline prevalence of the 3 forms of housing insecurity was 1371 (17.2%) for poor housing affordability, 1579 (19.5%) for poor housing quality, and 813 (8.9%) for poor neighborhood quality (Table). The mean and median follow-up times are provided in eTable 4 in Supplement 1. As shown in Figure 2 and eFigure 2 in Supplement 1, the cumulative incidence of frailty, disability, and dementia, as well as cumulative mortality, was consistently higher among participants with each form of housing insecurity compared with those in the adequate group.

**Table.** Baseline Characteristics of Community-Living National Health and Aging Trends Study Participants

Variables	Participants, No. (weighted %)^a^
Unweighted total participants	7499
Weighted total population, No.	40 728 543
Sex	
Male	3164 (44.7)
Female	4335 (55.3)
Age group, y	
65-69	1045 (29.3)
70-74	1755 (26.7)
75-79	1575 (18.7)
80-84	1399 (12.6)
85-89	1016 (8.1)
≥90	709 (4.6)
Race and ethnicity^b^	
Hispanic	441 (7.0)
Non-Hispanic Black	1545 (8.1)
Non-Hispanic White	5105 (77.8)
Other	408 (7.1)
Education	
Less than high school	1600 (16.7)
High school or equivalent	1970 (25.2)
Beyond high school	1575 (22.5)
College graduate or beyond	2171 (32.6)
Missing	183 (3.0)
Medicaid eligible	
No	6436 (87.9)
Yes	1063 (12.1)
Annual household income, $^c^	
≥14 700	6000 (84.6)
<14 700	1499 (15.4)
Smoking status	
Noncurrent smoker	6932 (91.7)
Current smoker	554 (8.2)
Missing	13 (0.1)
No. of chronic conditions, mean (SD)^d^	2.5 (1.5)
Housing affordability	
Adequate	5630 (75.5)
Poor	1371 (17.2)
Missing	498 (7.3)
Housing quality	
Adequate	5352 (71.8)
Poor	1579 (19.5)
Missing	568 (8.7)
Neighborhood quality	
Adequate	6529 (88.7)
Poor	813 (8.9)
Missing	157 (2.4)

^a^ Data are presented as unweighted number of participants and weighted percentages to represent the population of Medicare beneficiaries aged 65 years or older in 2015.

^b^ Race and ethnicity were self-reported, and the other category includes participants who reported their race and ethnicity as Asian, American Indian, Native Hawaiian, Other Pacific Islander, other, do not know, or more than 1 race and ethnicity.

^c^ Household income refers to the total annual income of the participant and their cohabiting spouse or partner. Missing values were imputed by the National Health and Aging Trends Study. The cutoff of $14 700 represents the threshold for the lowest income quintile.

^d^ Includes 9 self-reported, physician-diagnosed chronic conditions, including heart attack, heart disease, high blood pressure, arthritis, osteoporosis, diabetes, lung disease, stroke, and cancer.

**Figure 3.**
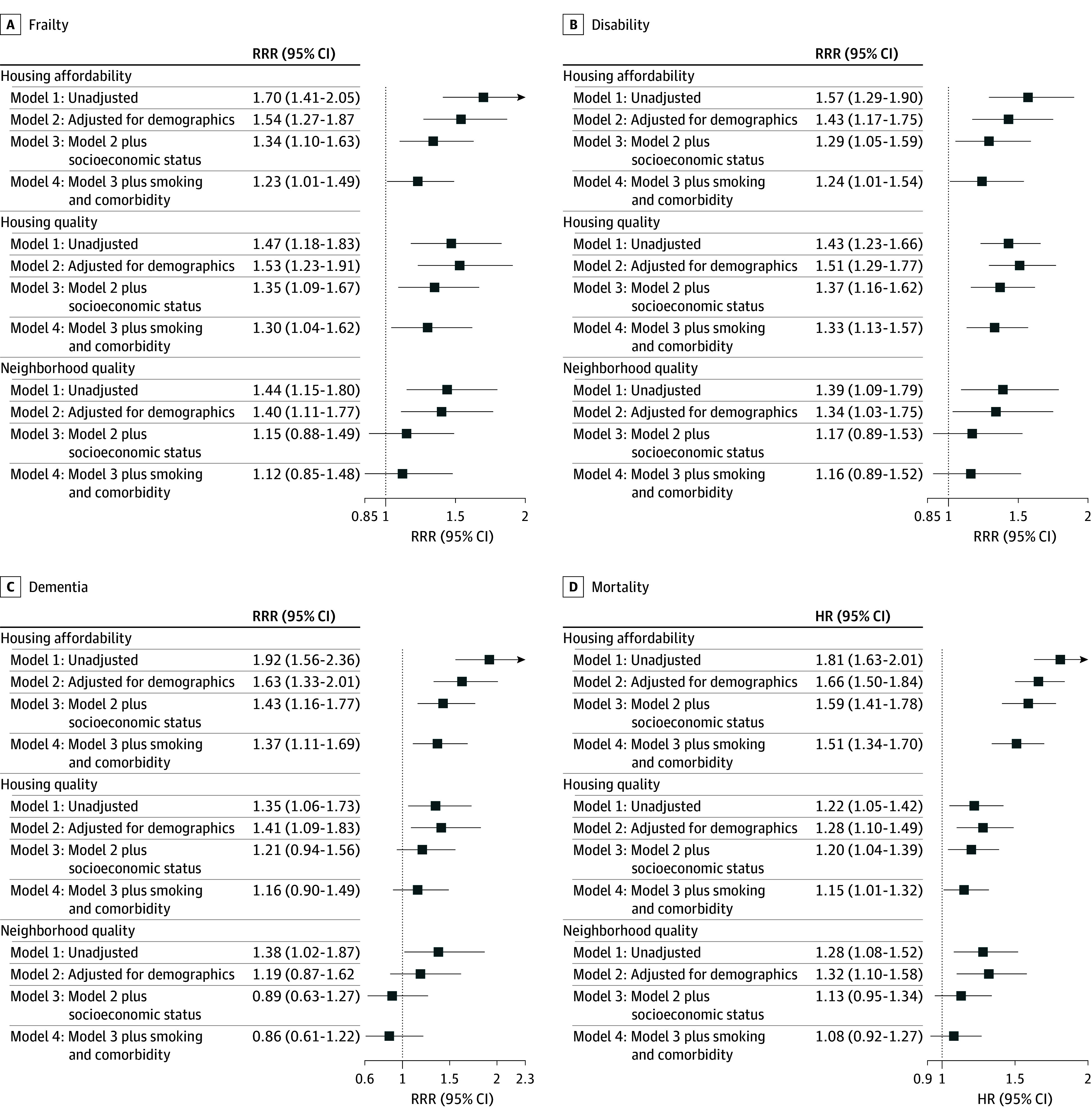
Dot Plots of Relative Risk Ratios (RRRs) and Hazard Ratios (HRs) for 5-Year Incident Geriatric Conditions and Mortality According to Housing Insecurity RRRs and 95% CIs for geriatric conditions were generated from discrete-time cause-specific hazards models accounting for the competing risk of death, whereas HRs and 95% CIs for mortality were generated from time-varying Cox regression models. Analytic weights provided by National Health and Aging Trends Study were applied to account for differential probabilities of selection and nonresponse. Model 1 was unadjusted, corresponding to the cumulative incidence and/or mortality curves in Figure 2 and eFigure 2 in Supplement 1. Model 2 was adjusted for demographic characteristics, including sex, age, and race and ethnicity. Model 3 included the covariates in model 2 plus socioeconomic status (education, Medicaid eligibility, and household income). Model 4 included the covariates in model 3 plus smoking status and comorbidity.

**Figure 4.**
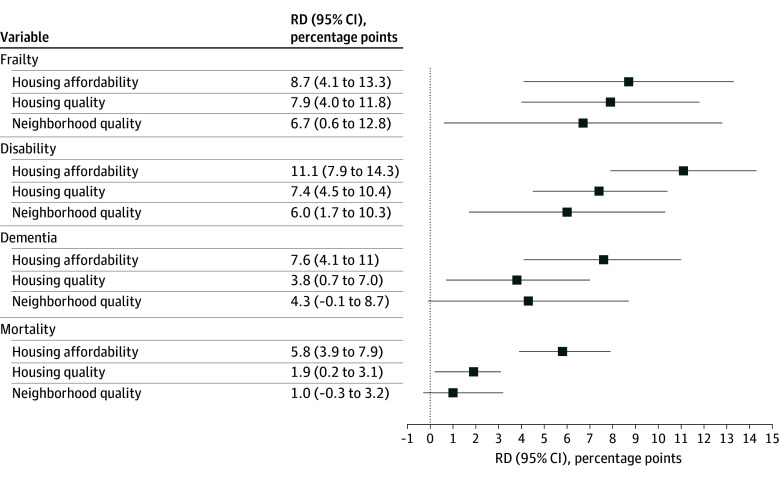
Dot Plot of Adjusted Risk Differences (RDs) for 5-Year Incident Geriatric Conditions and Mortality According to Housing Insecurity All estimates were adjusted for sex, age, race and ethnicity, education, Medicaid eligibility, household income, smoking status, and comorbidity, based on discrete-time cause-specific hazard models accounting for the competing risk of death. The 95% CIs were calculated using 300 bootstrap replications. Analytic weights provided by National Health and Aging Trends Study were applied to account for differential probabilities of selection and nonresponse.

Figure 3 provides the RRRs and HRs for each outcome according to the 3 forms of housing insecurity. After adjustment for age, sex, race and ethnicity, education, Medicaid eligibility, household income, smoking status, and comorbidity, poor housing affordability was significantly associated with increased risks of developing frailty (RRR, 1.23; 95% CI, 1.01-1.49), disability (RRR, 1.24; 95% CI, 1.01-1.54), and dementia (RRR, 1.37; 95% CI, 1.11-1.69), as well as mortality (HR, 1.51; 95% CI, 1.34-1.70). Similarly, poor housing quality was significantly associated with higher risks of developing frailty (RRR, 1.30; 95% CI, 1.04-1.62) and disability (RRR, 1.33; 95% CI, 1.13-1.57), and a higher risk of mortality (HR, 1.15; 95% CI, 1.01-1.32), but not dementia (RRR, 1.16, 95% CI, 0.90-1.49). For poor neighborhood quality, none of the associations was statistically significant in the fully adjusted models. E-values for the fully adjusted models ranged from 1.76 (frailty) to 2.39 (mortality) for housing affordability and from 1.57 (mortality) to 1.99 (disability) for housing quality (eTable 5 in Supplement 1), suggesting that a moderate to strong unmeasured confounder would be necessary to negate the observed associations. Results remained largely unchanged after applying multiple imputation and further adjustment for the GERiCounty, respectively (eTable 6 in Supplement 1). Figure 4 presents the risk differences (RDs) from the fully adjusted models, ranging from 1.9 percentage points (95% CI, 0.2%-3.1%) for housing quality with mortality to 11.1 percentage points (95% CI, 7.9%-14.3%) for housing affordability with disability.

## Discussion

In this nationally representative cohort of community-living older persons, poor housing affordability was significantly associated with higher subsequent risks of frailty, disability, dementia, and mortality after adjustment for age, sex, race and ethnicity, education, Medicaid eligibility, household income, smoking status, and comorbidity. Poor housing quality was significantly associated with higher risks of frailty, disability, and mortality, but not dementia. In contrast, poor neighborhood quality was not associated with any outcome. Taken together, these findings underscore housing insecurity as a potentially underrecognized SDOH with important and clinically relevant implications for the health and well-being of older persons.

As the US population ages, the number of older persons living with housing insecurity is expected to rise substantially.^2,3,40^ Prior housing insecurity studies of older persons have focused primarily on homelessness,^41,42^ have been limited to specific subpopulations (eg, low-income and moderate-income renters),^10^ have used cross-sectional designs,^43,44,45^ or are not nationally representative.^42,46^ None, to our knowledge, has comprehensively evaluated geriatric-specific conditions in relation to housing insecurity. Using data from a well-phenotyped and nationally representative cohort of community-living older US individuals, we were able to address each of these limitations and, in turn, evaluate the associations between 3 key forms of housing insecurity and the development of frailty, disability, and dementia, as well as mortality.

Our findings are consistent with prior research linking unaffordable housing to adverse health outcomes in low-income to middle-income older renters, including nursing home placement and functional impairment.^10,47^ Financial trade-off is one potential mechanism underlying our findings on poor housing affordability. Specifically, high housing costs may limit older persons’ spending on health care, basic necessities, and social or recreational activities.^10^ These financial constraints attributable to high housing costs may lead to unmet medical needs, reliance on lower-quality essential goods, and reduced social support networks—all of which are critical to quality of life and well-being in older persons.^10^ For example, prior research has shown that persons living in unaffordable housing are more likely to delay physician visits or report cost-related nonadherence to medications and care.^48,49^ Our findings on housing affordability not only highlight the critical role of SDOH^50^ but also provide valuable insights for geriatric clinical practice. For instance, the recently released Age-Friendly Hospital metrics by the Centers for Medicare & Medicaid Services^51^ have included several SDOH indicators—such as social isolation and economic insecurity—highlighting the potential value of incorporating housing affordability into future care models.

Several potential mechanisms may underlie our findings on poor housing quality. First, participants living in housing with tripping hazards may be more likely to experience falls and injuries, directly increasing the risk of frailty, disability, and mortality.^44,52,53^ Second, poor housing quality may also expose participants to a range of other environmental hazards. For example, broken windows or open holes may lead to poor insulation and increased noise, and interior items in disrepair may allow dust, mold, and other allergens to accumulate.^54,55^ These exposures may cause or exacerbate cardiovascular, mental health, and respiratory conditions, thereby potentially increasing vulnerability to the development of frailty, disability, and mortality.^44,56,57^ Moreover, prior research has shown that poor housing quality, including uneven exterior walking surfaces or broken steps leading to the home, is associated with reduced physical activity in older persons,^58,59^ an important protective factor against these adverse outcomes. Although modest, the adjusted risk difference in mortality risk between the poor-quality and adequate-quality housing groups (1.9%) corresponds to approximately 150 000 additional deaths over 5 years among persons living in poor-quality housing, highlighting the potential population-level relevance of even small absolute differences.

Neighborhood quality also plays an important role in ensuring that older persons can live in environments that are safe, well-maintained, and favorable to good health,^60^ which aligns with broader definitions of housing insecurity.^1,61^ Nonetheless, a recent meta-analysis found no consistent evidence linking neighborhood quality to health outcomes.^62^ Our prior work has found that older persons residing in disadvantaged counties, measured by contextual-level SDOH indices, had a higher incidence of frailty, disability, and dementia over a 5-year period.^25^ While such contextual-level indices provide valuable insights into structural and environmental determinants of health, they may fail to capture the individual lived experiences of housing insecurity. An important strength of our current study is that both housing and neighborhood quality were assessed by trained interviewers at the individual level, providing a more direct and objective assessment of participants’ true living conditions. In the current study, we found that poor neighborhood quality was no longer significantly associated with any outcomes after adjustment for socioeconomic status, suggesting that the associations may be largely attributable to socioeconomic status.

### Strengths and Limitations

This study has several strengths, including its prospective cohort design, a nationally representative sample, collection of 3 time-varying, NHATS-validated forms of housing insecurity,^2^ complete ascertainment of mortality through linked Medicare records, and 3 well-phenotyped, NHATS-validated geriatric outcomes.^23,24,25^ Despite these strengths, several limitations should be considered when interpreting the findings. First, as an observational study, the reported associations cannot be interpreted as causal.^36,37^ Second, NHATS does not include indicators of housing instability, such as forced moves (eg, eviction), frequent moves (eg, ≥2 in a year), or foreclosure.^1^ Future research should address this dimension of housing insecurity as relevant data become available. Third, the incidence of frailty was likely underestimated in 2020 because only 3 of the 5 criteria could be assessed during the COVID-19 pandemic. Fourth, because NHATS included participants from only the 48 contiguous US states, our findings may not be generalizable to older persons from Alaska, Hawaii, or Puerto Rico.

## Conclusions

In this cohort study of community-living older US individuals, housing insecurity—particularly poor housing affordability and poor housing quality—was significantly associated with the development of geriatric conditions and mortality over a 5-year follow-up period. These findings underscore the need to recognize housing insecurity as an important and clinically relevant SDOH in older persons.

## Notes

Supplement 1. eTable 1. Concordance of Housing Affordability Between Adjacent NHATS Rounds eTable 2. Concordance of Housing Quality Between Adjacent NHATS Rounds eTable 3. Concordance of Neighborhood Quality Between Adjacent NHATS Rounds eTable 4. Follow-up Times (Years) by Outcome eTable 5. E-Values for Main Analysis eTable 6. Sensitivity Analyses eFigure 1. Conceptual Framework Linking Housing Insecurity with Incident Geriatric Conditions and Mortality in Older Persons eFigure 2. Cumulative Mortality Over 5 Years for Three Forms of Housing Insecurity eReferences.

Supplement 2. Data Sharing Statement
