# Supplementary material for: Housing Insecurity, Incident Geriatric Conditions, and Mortality in Community-Living Older Persons
Source: JAMA Netw Open. 2026 May 1;9(5):e269335. doi: 10.1001/jamanetworkopen.2026.9335 (PMC13135211; doi:10.1001/jamanetworkopen.2026.9335)
Supplement: Supplement 1. — eTable 1. Concordance of Housing Affordability Between Adjacent NHATS Rounds eTable 2. Concordance of Housing Quality Between Adjacent NHATS Rounds eTable 3. Concordance of Neighborhood Quality Between Adjacent NHATS Rounds eTable 4. Follow-up Times (Years) by Outcome eTable 5. E-Values for Main Analysis eTable 6. Sensitivity Analyses eFigure 1. Conceptual Framework Linking Housing Insecurity with Incident Geriatric Conditions and Mortality in Older Persons eFigure 2. Cumulative Mortality Over 5 Years for Three Forms of Housing Insecurity eReferences. [file jamanetwopen-e269335-s001.pdf]

Supplemental Online Content

Wang Y, Davis-Plourde K, Vander Wyk B, Paredes LG, Gill TM, Becher RD. Housing insecurity, incident geriatric conditions, and mortality in community-living older persons. *JAMA Netw Open.* 2026;9(4):e269335. doi:10.1001/jamanetworkopen.2026.9335

eTable 1. Concordance of Housing Affordability Between Adjacent NHATS Rounds

eTable 2. Concordance of Housing Quality Between Adjacent NHATS Rounds

eTable 3. Concordance of Neighborhood Quality Between Adjacent NHATS Rounds

eTable 4. Follow-up Times (Years) by Outcome

eTable 5. E-Values for Main Analysis

eTable 6. Sensitivity Analyses

eFigure 1. Conceptual Framework Linking Housing Insecurity with Incident Geriatric Conditions and Mortality in Older Persons

eFigure 2. Cumulative Mortality Over 5 Years for Three Forms of Housing Insecurity

eReferences.

This supplemental material has been provided by the authors to give readers additional information about their work.

© 2026 Wang Y et al. *JAMA Network Open*.

eTable 1. Concordance of Housing Affordability Between Adjacent NHATS Rounds[a]

Round 5	Round 6			Agreement	Weighted Kappa[b], 95% CI
	Adequate	Poor	Total		
Adequate	4529	189	4718	94.3%	0.82 (0.79-0.85)
Poor	143	937	1080		
Total	4672	1126	5798		
Round 6	Round 7			Agreement	Weighted Kappa[b], 95% CI
	Adequate	Poor	Total		
Adequate	3893	292	4185	88.8%	0.64 (0.60-0.67)
Poor	286	665	951		
Total	4179	957	5136		
Round 7	Round 8			Agreement	Weighted Kappa[b], 95% CI
	Adequate	Poor	Total		
Adequate	3605	149	3754	95.0%	0.86 (0.83-0.88)
Poor	80	724	804		
Total	3685	873	4558		
Round 8	Round 9			Agreement	Weighted Kappa[b], 95% CI
	Adequate	Poor	Total		
Adequate	3143	226	3369	90.2%	0.69 (0.65-0.72)
Poor	178	587	765		
Total	3321	813	4134		

Abbreviations: NHATS, National Health and Aging Trends Study; CI, confidence interval.
[a] Unless otherwise stated, all values are unweighted.
[b] Analytic weights provided by NHATS were applied to account for differential probabilities of selection and non-response.

© 2026 Wang Y et al. *JAMA Network Open*.

eTable 2. Concordance of Housing Quality Between Adjacent NHATS Rounds[a]

Round 5	Round 6			Agreement	Weighted Kappa[b], 95% CI
	Adequate	Poor	Total		
Adequate	3952	521	4473	80.6%	0.45 (0.42-0.49)
Poor	596	694	1290		
Total	4548	1215	5763		
Round 6	Round 7			Agreement	Weighted Kappa[b], 95% CI
	Adequate	Poor	Total		
Adequate	3573	490	4063	81.2%	0.44 (0.39-0.48)
Poor	468	574	1042		
Total	4041	1064	5105		
Round 7	Round 8			Agreement	Weighted Kappa[b], 95% CI
	Adequate	Poor	Total		
Adequate	3201	404	3605	81.7%	0.44 (0.39-0.50)
Poor	428	506	934		
Total	3629	910	4539		
Round 8	Round 9			Agreement	Weighted Kappa[b], 95% CI
	Adequate	Poor	Total		
Adequate	2890	387	3277	79.9%	0.36 (0.32-0.41)
Poor	433	373	806		
Total	3323	760	4083		

Abbreviations: NHATS, National Health and Aging Trends Study; CI, confidence interval.
[a] Unless otherwise stated, all values are unweighted.
[b] Analytic weights provided by NHATS were applied to account for differential probabilities of selection and non-response.

© 2026 Wang Y et al. *JAMA Network Open*.

eTable 3. Concordance of Neighborhood Quality Between Adjacent NHATS Rounds[a]

Round 5	Round 6			Agreement	Weighted Kappa[b], 95% CI
	Adequate	Poor	Total		
Adequate	5154	338	5492	89.1%	0.42 (0.36-0.48)
Poor	334	344	678		
Total	5488	682	6170		
**Round 6**	**Round 7**			**Agreement**	**Weighted Kappa[b], 95% CI**
	Adequate	Poor	Total		
Adequate	4529	280	4809	89.3%	0.42 (0.36-0.47)
Poor	295	281	576		
Total	4824	561	5385		
**Round 7**	**Round 8**			**Agreement**	**Weighted Kappa[b], 95% CI**
	Adequate	Poor	Total		
Adequate	4074	216	4290	89.8%	0.39 (0.32-0.46)
Poor	272	214	486		
Total	4346	430	4776		
**Round 8**	**Round 9**			**Agreement**	**Weighted Kappa[b], 95% CI**
	Adequate	Poor	Total		
Adequate	3734	170	3904	91.3%	0.45 (0.38-0.52)
Poor	202	179	381		
Total	3936	349	4285		

Abbreviations: NHATS, National Health and Aging Trends Study; CI, confidence interval.
[a] Unless otherwise stated, all values are unweighted,
[b] Analytic weights provided by NHATS were applied to account for differential probabilities of selection and non-response.

© 2026 Wang Y et al. *JAMA Network Open*.

eTable 4. Follow-up Times (Years) by Outcome

Outcome	Mean (SD)	Median (IQR)
Frailty	3.8 (1.9)	5.0 (2.0–5.0)
Disability	4.0 (1.9)	5.0 (4.0–5.0)
Dementia	3.6 (1.9)	5.0 (2.0–5.0)
Mortality	5.5 (1.5)	5.5 (4.6–5.5)

Abbreviations: SD, standard deviation; IQR, interquartile range.
Reversed Kaplan–Meier method was used to estimate mean and median for follow-up time.

© 2026 Wang Y et al. *JAMA Network Open*.

eTable 5. E-Values for Main Analysis

Outcome	Effect Size (RRR/HR)	E-value (lower 95% CI)
Frailty		
Housing Affordability	1.23 (1.01-1.49)	1.76 (1.11)
Housing Quality	1.30 (1.04-1.62)	1.92 (1.24)
Neighborhood Quality	1.12 (0.85-1.48)	-
Disability		
Housing Affordability	1.24 (1.01-1.54)	1.79 (1.11)
Housing Quality	1.33 (1.13-1.57)	1.99 (1.51)
Neighborhood Quality	1.16 (0.89-1.52)	-
Dementia		
Housing Affordability	1.37 (1.11-1.69)	2.08 (1.46)
Housing Quality	1.16 (0.90-1.49)	-
Neighborhood Quality	0.86 (0.61-1.22)	-
Mortality		
Housing Affordability	1.51 (1.34-1.70)	2.39 (2.01)
Housing Quality	1.15 (1.01-1.32)	1.57 (1.11)
Neighborhood Quality	1.08 (0.92-1.27)	-

Abbreviations: RRR, relative risk ratio; HR, hazard ratio; CI, confidence interval.
Frailty, disability, and dementia were analyzed using discrete cause-specific hazards models, yielding RRRs, whereas mortality was analyzed using Cox proportional hazards models, yielding HRs. All estimates were adjusted for age, sex, race/ethnicity, education, Medicaid eligibility, household income, smoking status, and comorbidity. E-values were calculated and reported only for statistically significant estimates presented in Figure 3.

© 2026 Wang Y et al. *JAMA Network Open*.

eTable 6. Sensitivity Analyses

Sensitivity Analysis (SA)	Frailty RRR (95% CI)	Disability RRR (95% CI)	Dementia RRR (95% CI)	Mortality HR (95% CI)
SA 1. Multiple imputation				
Housing affordability	1.25 (1.02-1.53)	1.21 (1.02-1.44)	1.35 (1.11-1.65)	1.47 (1.32-1.62)
Housing quality	1.31 (1.05-1.63)	1.33 (1.14-1.56)	1.18 (0.92-1.52)	1.15 (1.00-1.31)
Neighborhood quality	1.07 (0.82-1.39)	1.13 (0.86-1.47)	0.89 (0.63-1.24)	1.07 (0.94-1.23)
SA 2. Models additionally accounted for county-level disadvantage				
Housing affordability	1.27 (1.05-1.54)	1.30 (1.05-1.62)	1.41 (1.15-1.74)	1.41 (1.18-1.69)
Housing quality	1.31 (1.05-1.64)	1.35 (1.14-1.60)	1.13 (0.87-1.48)	1.24 (1.10-1.41)
Neighborhood quality	1.10 (0.83-1.44)	1.18 (0.90-1.54)	0.85 (0.59-1.21)	1.06 (0.86-1.31)

Abbreviations: RRR, relative risk ratio; HR, hazard ratio; CI, confidence interval.
Notes: All analyses applied NHATS weights to account for differential probabilities of selection and non-response.
Sensitivity Analysis 1: We applied multiple imputation by chained equations (10 imputations) to address missing data on education, smoking status, and housing insecurity, incorporating all covariates. All models were adjusted for sex, age, race and ethnicity, educational attainment, Medicaid eligibility, household income, smoking status, and comorbidity.
Sensitivity Analysis 2: All models were adjusted for sex, age, race and ethnicity, education, Medicaid eligibility, household income, smoking status, comorbidity, and county-level disadvantage, measured by the Geriatric Index of County-Level Multi-Dimensional Contextual Disadvantage (GERiCounty).

© 2026 Wang Y et al. *JAMA Network Open*.

eFigure 1. Conceptual Framework Linking Housing Insecurity with Incident Geriatric Conditions and Mortality in Older Persons

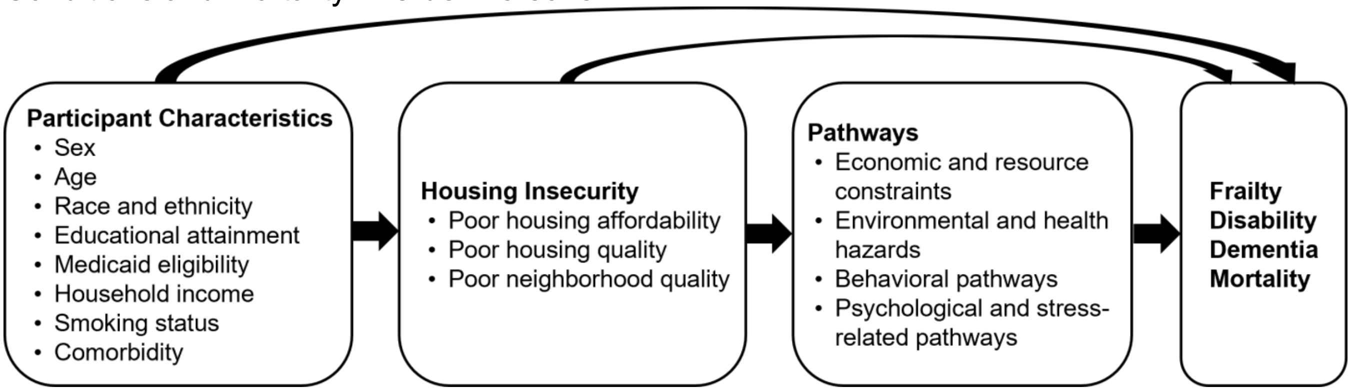

Notes: Based on a comprehensive review of the literature,[1-6] we developed this conceptual framework to illustrate the potential confounders and pathways linking housing insecurity with incident geriatric conditions and mortality in older persons. To account for both omitted variable bias and overadjustment,[7,8] the final fully adjustment set included sex, age, race and ethnicity, educational attainment, Medicaid eligibility, household income, smoking status, and comorbidity. Comorbidity was measured as the count of 9 self-reported, physician-diagnosed chronic conditions, including heart attack, heart disease, high blood pressure, arthritis, osteoporosis, diabetes, lung disease, stroke, and cancer.[9] Drawing on prior literature, we hypothesize that the association between housing insecurity and the outcomes may be mediated through economic and resource constraints (e.g., reduced healthcare spending, limited basic necessities, cost-related medication nonadherence, relocation to more disadvantaged neighborhoods),[10-13] environmental and health hazards (e.g., increased risk of falls, injuries, and limitations in physical function, vulnerability to acute and chronic illnesses),[14-20] behavioral pathways (e.g., lack of physical activity, limited social engagement),[21-24] and psychological and stress-related pathways (e.g., elevated stress, anxiety, depression, weakened social support, marital disagreement),[1,25-27] although these pathways were not evaluated in this study. The potential mediators along the pathways are not mutually exclusive and may interact or reinforce with one another. The full complexity of these interrelated pathways is not captured in this conceptual framework.

© 2026 Wang Y et al. *JAMA Network Open*.

eFigure 2. Cumulative Mortality Over 5 Years for Three Forms of Housing Insecurity

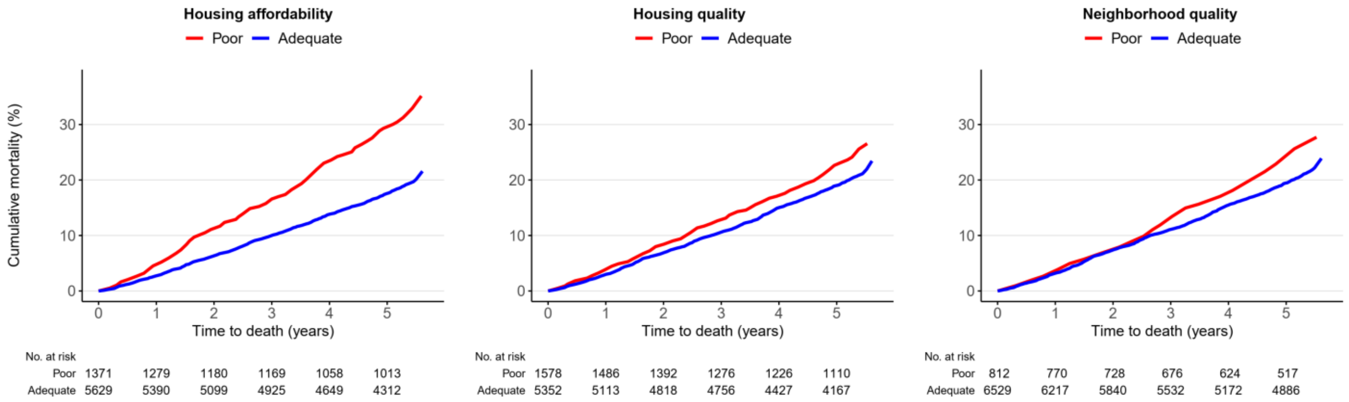

Abbreviations: No, number. Analytic weights provided by NHATS were applied to account for differential probabilities of selection and non-response. The number at risk represents unweighted values. To meet the minimizing-disclosure-risk rule of the NIA Data LINKAGE Program, certain intervals were collapsed to ensure that each contained at least 11 unweighted deaths.

© 2026 Wang Y et al. *JAMA Network Open*.

eReferences

1. Bhat AC, Fenelon A, Almeida DM. Housing insecurity pathways to physiological and epigenetic manifestations of health among aging adults: A conceptual model. *Front Public Health*. 2025;13:1485371. doi:10.3389/fpubh.2025.1485371
2. Howden-Chapman P, Bennett J, Edwards R, Jacobs D, Nathan K, Ormandy D. Review of the impact of housing quality on inequalities in health and well-being. *Annu Rev Public Health*. 2023;44:233-254. doi:10.1146/annurev-publhealth-071521-111836
3. O'Brien DT, Farrell C, Welsh BC. Broken (windows) theory: A meta-analysis of the evidence for the pathways from neighborhood disorder to resident health outcomes and behaviors. *Soc Sci Med*. 2019;228:272-292. doi:10.1016/j.socscimed.2018.11.015
4. Heller C, Haak M, Schmidt SM, et al. The relationship between physical housing characteristics, housing accessibility and different aspects of health among community-dwelling older people: A systematic review. *J Aging Health*. 2024;36(1-2):120-132. doi:10.1177/08982643231175367
5. Swope CB, Hernandez D. Housing as a determinant of health equity: A conceptual model. *Soc Sci Med*. 2019;243:112571. doi:10.1016/j.socscimed.2019.112571
6. Bentley R, Mason K, Jacobs D, et al. Housing as a social determinant of health: A contemporary framework. *Lancet Public Health*. 2025;10(10):e855-e864. doi:10.1016/S2468-2667(25)00142-2
7. Schisterman EF, Cole SR, Platt RW. Overadjustment bias and unnecessary adjustment in epidemiologic studies. *Epidemiology*. 2009;20(4):488-95. doi:10.1097/EDE.0b013e3181a819a1
8. Cinelli C, Forney A, Pearl J. A crash course in good and bad controls. *Sociol Method Res*. 2024;53(3):1071-1104. doi:10.1177/00491241221099552
9. Falvey JR, Cohen AB, O'Leary JR, Leo-Summers L, Murphy TE, Ferrante LE. Association of social isolation with disability burden and 1-year mortality among older adults with critical illness. *JAMA Intern Med*. 2021;181(11):1433-1439. doi:10.1001/jamainternmed.2021.5022
10. Hernandez M, Wong R, Yu X, Mehta N. In the wake of a crisis: Caught between housing and healthcare. *SSM Popul Health*. 2023;23:101453. doi:10.1016/j.ssmph.2023.101453
11. Moulton S, Rhodes A, Haurin D, Loibl C. Managing the onset of a new disease in older age: Housing wealth, mortgage borrowing, and medication adherence. *Soc Sci Med*. 2022;314:115437. doi:10.1016/j.socscimed.2022.115437
12. Jenkins Morales M, Robert SA. The effects of housing cost burden and housing tenure on moves to a nursing home among low- and moderate-income older adults. *Gerontologist*. 2020;60(8):1485-1494. doi:10.1093/geront/gnaa052
13. Evans M. The unequal housing and neighborhood outcomes of displaced movers. *J Urban Aff*. 2020;43(2):1214-1234. doi:10.1080/07352166.2020.1730697
14. Krieger J, Higgins DL. Housing and health: Time again for public health action. *Am J Public Health*. 2002;92(5):758-68. doi:10.2105/ajph.92.5.758
15. Garcia-Esquinas E, Perez-Hernandez B, Guallar-Castillon P, Banegas JR, Ayuso-Mateos JL, Rodriguez-Artalejo F. Housing conditions and limitations in physical function among older adults. *J Epidemiol Community Health*. 2016;70(10):954-60. doi:10.1136/jech-2016-207183
16. Cheng H, Shao L, Xie T, et al. Housing quality and related housing environmental factors associated with falls in older adults: Results from the longitudinal ageing study in india. *Age Ageing*. 2025;54(9)doi:10.1093/ageing/afaf260
17. Li S, Ren L, Hu Y, et al. Associations between housing quality and sarcopenia among older adults: Evidence from china and india. *J Nutr Health Aging*. 2025;29(2):100449. doi:10.1016/j.jnha.2024.100449
18. Pollack CE, Griffin BA, Lynch J. Housing affordability and health among homeowners and renters. *Am J Prev Med*. 2010;39(6):515-21. doi:10.1016/j.amepre.2010.08.002
19. Rhodes A, Moulton S, Loibl C, Haurin D, Joseph J. Mortgage borrowing and chronic disease outcomes in older age: Evidence from biomarker data in the health and retirement study. *J Gerontol B Psychol Sci Soc Sci*. 2024;79(7)doi:10.1093/geronb/gbae066

© 2026 Wang Y et al. *JAMA Network Open*.

20. Okoye SM, Samuel LJ, Fabius C, et al. Home and neighborhood context of falls among black and white older Americans. *J Aging Health*. 2021;33(9):721-731. doi:10.1177/08982643211009436
21. Stahre M, VanEenwyk J, Siegel P, Njai R. Housing insecurity and the association with health outcomes and unhealthy behaviors, washington state, 2011. *Prev Chronic Dis*. 2015;12:E109. doi:10.5888/pcd12.140511
22. Kwon KY, Kim J. Housing hardship and health: Longitudinal evidence of the mediating role of health behaviors. *Soc Sci Med*. 2025;366:117702. doi:10.1016/j.socscimed.2025.117702
23. Kotlarczyk MP, Hergenroeder AL, Gibbs BB, Cameron FA, Hamm ME, Brach JS. Personal and environmental contributors to sedentary behavior of older adults in independent and assisted living facilities. *Int J Environ Res Public Health*. 2020;17(17)doi:10.3390/ijerph17176415
24. Azim FT, Ariza-Vega P, Gardiner PA, Ashe MC. Indoor built environment and older adults' activity: A systematic review. *Can J Aging*. 2023;42(2):241-258. doi:10.1017/S0714980822000241
25. Evans GW, Kantrowitz E, Eshelman P. Housing quality and psychological well-being among the elderly population. *J Gerontol B Psychol Sci Soc Sci*. 2002;57(4):P381-3. doi:10.1093/geronb/57.4.p381
26. Ascigil E, Selcuk E, Gunaydin G, Ong AD. Integrating models of marital functioning to understand the mental health consequences of the great recession. *J Soc Pers Relat*. 2020;37(7):2118-2135. doi:10.1177/0265407520918938
27. Pevalin DJ, Reeves A, Baker E, Bentley R. The impact of persistent poor housing conditions on mental health: A longitudinal population-based study. *Prev Med*. 2017;105:304-310. doi:10.1016/j.ypmed.2017.09.020

© 2026 Wang Y et al. *JAMA Network Open*.
