# Supplementary material for: Housing Insecurity, Incident Geriatric Conditions, and Mortality in Community-Living Older Persons
Source: JAMA Netw Open. 2026 May 1;9(5):e269335. doi: 10.1001/jamanetworkopen.2026.9335 (PMC13135211; doi:10.1001/jamanetworkopen.2026.9335)
Supplement: Supplement 2. — Data Sharing Statement [file jamanetwopen-e269335-s002.pdf]

Data Sharing Statement

Wang. Housing Insecurity, Incident Geriatric Conditions, and Mortality in Community-Living Older Persons. *JAMA Netw Open*. Published May 01, 2026.
doi:10.1001/jamanetworkopen.2026.9335

Data
Data available: No
